# Perceived value, structural barriers, and academic burnout under the “hard science vs. soft skills” paradox: a cross-sectional study among Chinese medical students

**DOI:** 10.3389/fpsyg.2026.1839448

**Published:** 2026-09-03

**Authors:** Le Li, Yingli Xue, Jianan Yang, Ting Xue, Na Zhao

**Affiliations:** School of Foreign Studies, Xi'an Medical University, Xi'an, China

**Keywords:** academic burnout, canonical correlation analysis, medical humanities, medical students, perceived value, structural barriers

## Abstract

**Introduction:**

Medical education frequently prioritizes biomedical knowledge over humanistic training, a hierarchy that may be associated with academic burnout. While the tension between “hard science” and “soft skills” is a recognized global challenge, empirical evidence regarding how students' specific cognitive appraisals, especially perceived value and structural barriers, relate to burnout. This study examined the multivariate relationship between these appraisal components and academic burnout among clinical medical students in China.

**Methods:**

A cross-sectional survey was conducted among 165 undergraduate clinical medical students. Participants completed the Perceived Value Scale, Perceived Barriers Scale, and a modified Academic Burnout Scale. The adapted perceived value and perceived barriers instruments were documented through item-domain mapping, exploratory factor analysis/principal-component extraction, and internal consistency evidence. Canonical correlation analysis (CCA) was used to examine associations between the cognitive appraisal set and burnout dimensions.

**Results:**

Students reported high perceived value of medical humanities (*M* = 4.25) despite moderate structural barriers (*M* = 3.06). Perceived value and perceived barriers were not significantly correlated (*r* = −0.075, *p* > 0.05), indicating cognitive decoupling between valuing humanities education and perceiving structural obstacles. The first canonical function was significant (*r*_*c*_ = 0.502, *p* < 0.001). After distinguishing canonical weights from structure coefficients, the function indicated that higher perceived value and lower perceived barriers were associated with a more favorable burnout profile. Within the burnout set, cynicism showed the strongest structure coefficient (−0.796), followed by academic efficacy (0.682) and emotional exhaustion (−0.678), suggesting that structural barriers were most closely aligned with psychological detachment from learning.

**Conclusion:**

Perceived value and structural barriers appear to represent related but distinct educational appraisals. The findings suggest that increasing recognition of medical humanities may be insufficient if structural learning costs remain high. Medical education reform may therefore benefit from shifting from an additive model to an integrative model that embeds humanities into clinical learning contexts, reduces perceived barriers, and supports professional development.

## 1 Introduction

The evolving medical paradigm—shifting from a traditional biomedical model to a biopsychosocial framework (Engel, 1977)—has redefined the objectives of modern medical education. Beyond the mastery of “hard science” disciplines like anatomy and pathology, contemporary medical education increasingly prioritizes the cultivation of humanistic qualities—such as empathy, ethical discernment, and professional resilience—as essential components of a physician's professional identity (Wald, 2020; Simou, 2025). Medical humanities curricula are strategically positioned as the essential bridge between technical proficiency and humanistic care. However, within an educational ethos often dominated by technical rationality, a persistent tension exists between these two domains, particularly in high-stakes, competitive academic environments (Iorio et al., 2022). This “hard science” vs. “soft skills” paradox does more than complicate curriculum design; it profoundly shapes students' psychological adaptation and academic experiences. Recent studies further support this direction. For instance, integrating medical humanities into emergency skill-training simulations has been reported to improve educational outcomes, ethical literacy, and participant satisfaction (Guo et al., 2025). Additionally, a longitudinal undergraduate medical-humanities programme aligned with the InspirE5 framework has been proposed as a model for integrating empathy, ethical reflection, and critical thinking across medical training (Coronado-Vazquez et al., 2025).

Academic burnout represents a critical mental health challenge within this professional training pipeline. It is conceptualized as a psychological syndrome arising from protracted academic stress, manifested through three key dimensions: emotional exhaustion, cynicism (or depersonalization), and a diminished sense of academic efficacy (Maslach and Leiter, 2021). Recent meta-analyses indicate that nearly half of medical students worldwide experience some form of burnout, with medical students in China being particularly vulnerable due to excessive academic workloads and the rigor of the National Medical Licensing Examination (Chunming et al., 2017; Wang et al., 2023; Liu et al., 2023). The consequences of burnout are far-reaching, with possible implications for learning motivation, medical errors, compassion fatigue, and patient safety in future clinical practice (West et al., 2018). Beyond medical education, burnout has also been linked to adverse safety outcomes in other high-pressure occupational settings, including civil construction, which further highlights the broader relevance of stress- and burnout-related mechanisms while underscoring the need to interpret such evidence within its specific professional context (Tran et al., 2025).

Cognitive appraisal theories suggest that students' reactions to these pressures are governed by the dynamic interplay between individuals' valuing of a task and the perceived costs associated with it. Control-Value Theory (Pekrun, 2006) and the updated Expectancy-Value model of motivation (Eccles and Wigfield, 2020) both indicate that students' cognitive appraisal of their curriculum is related to their emotional responses and psychological state. When students attribute high “Perceived Value” to humanities, this positive appraisal may be associated with greater cognitive resilience and lower stress-related vulnerability (Pekrun, 2006). Conversely, what may be termed “Relative Cost” or “Perceived Barriers”—such as curricular overload and the encroachment on biomedical study time—represent a critical counter-force. Accumulating research suggests that high perceived costs may be related to maladaptive coping mechanisms and psychological entrapment within the rigid structures of technical training (Bartlett and Fowler, 2020).

Crucially, the multidimensional nature of academic burnout requires a more granular analysis of how these cognitive appraisals map onto specific symptoms. While emotional exhaustion is often a direct response to physical and mental workload, cynicism frequently emerges as a defensive detachment when individuals perceive a structural misalignment between their professional ideals and institutional realities (Maslach and Leiter, 2021). Within the intensely competitive Chinese medical education system, where “hard science” achievement is the primary currency for advancement, the structural barriers to humanities integration may be associated with a cynical hidden-curriculum perception that devalues non-technical skills. Despite extensive literature on pedagogical reforms, there is a distinct lack of research utilizing integrative statistical frameworks like Canonical Correlation Analysis (CCA) to deconstruct the multivariate synergy between cognitive appraisals and burnout dimensions. Specifically, it remains empirically unclear whether the cognitive dissonance of high value and high barriers is linked to cynicism, with potential implications for the professional empathy that medical schools strive to cultivate.

Existing medical-education research has documented the prevalence of burnout, the importance of empathy and learning environment, and the pedagogical value of medical humanities; however, fewer studies have examined how students simultaneously appraise the value and structural cost of humanities education, or how this paired appraisal relates to different burnout dimensions within a single multivariate model. Recent reviews of Chinese medical education likewise identify curriculum diversification and the integration of medical humanities as priorities for reform, reinforcing the need to examine barriers within the local educational system (Li and Chen, 2025).

Accordingly, the present study employs a cross-sectional survey to investigate clinical medical students' perceptions of humanities curricula. Moving beyond traditional univariate analysis, we utilize CCA to investigate the structural relationship between the cognitive variable set (value and barriers) and the burnout variable set (exhaustion, cynicism, and efficacy) as a whole. By examining these constructs simultaneously, this study aims to clarify the specific cognitive patterns that are associated with different burnout profiles, providing evidence-based insights for integrating medical humanities more effectively under the intense pressure of professional medical training.

Based on this framework, we specified three hypotheses: H1, perceived value and perceived barriers would represent related but distinguishable cognitive appraisals rather than opposite poles of a single continuum; H2, higher perceived value and lower perceived barriers would be associated with lower emotional exhaustion and cynicism and with higher academic efficacy; and H3, within the multivariate CCA solution, cynicism would show a particularly strong association with the value-barrier profile because it reflects psychological detachment from a curriculum perceived as misaligned with students' professional ideals.

## 2 Materials and methods

### 2.1 Study design and participants

A cross-sectional survey was conducted from November 19th to December 3rd, 2025, at Xi'an Medical University, Shaanxi Province, China. The study targeted undergraduate students majoring in Clinical Medicine, specifically those in their first and second years who were undergoing medical humanities coursework. The survey was administered via the online platform “Wenjuanxing” and distributed through class-based digital communication groups. Participation was voluntary and anonymous; eligible students were undergraduate clinical-medicine students currently exposed to medical-humanities coursework, and non-clinical majors were excluded before analysis. The recruitment route was therefore a class-based convenience survey within a single institution rather than a probability sample.

Strict quality control procedures were implemented to screen the raw data prior to analysis. Initially, 179 questionnaires were retrieved. To ensure sample homogeneity, we first excluded participants from non-clinical majors (*n* = 9). Subsequently, to rigorously control for response quality, a “straight-lining” detection method was applied; five questionnaires in which participants selected the identical response option for all Likert-scale items (indicating zero variance and lack of engagement) were identified as invalid and removed. Consequently, the final dataset consisted of 165 valid responses, yielding an effective response rate of 92.2%. The analytical sample included 39 freshmen (23.6%) and 126 sophomores (76.4%).

### 2.2 Instruments

General Information Questionnaire. A self-designed questionnaire was used to collect demographic information from the participants. Based on the study requirements, we collected data on grade (Freshman/Sophomore), major, gender, Grade Point Average (GPA), and general interest in medical humanities courses.

Perceived Value Scale (PV). Perceived task value, a core construct within the Expectancy-Value Theory, is defined as the subjective worth a learner attaches to a specific academic task—a critical factor that directly influences achievement behaviors and persistence (Wigfield and Eccles, 2000). To assess this construct within the specific context of medical education, we employed a Perceived Value Scale that was adapted to align with the core competencies of medical humanities curricula (Hojat, 2016; Huang et al., 2025). To ensure content validity, the initial item pool was refined through expert panel review and preliminary pilot testing. The review focused on whether each item matched the intended domain, used language understandable to junior medical students, and reflected the local curricular context of medical humanities education. The scale comprises 9 items explicitly categorized into four dimensions: Ethical Sensitivity (items 1–3), Empathy (items 4–5), Cultural Competence (items 6–7), and Personal Development & Communication (items 8–9). These dimensions represent cognitive alertness to moral conflicts, emotional resonance with patients, respect for diverse backgrounds, and practical utility for occupational stress, respectively. A representative item includes, “It makes me realize I should respect patients' values from different cultural backgrounds”. Participants rated each item on a 5-point Likert scale ranging from 1 (strongly disagree) to 5 (strongly agree). All items are positively worded. Because the present study used the scale as a global appraisal score, we further examined whether the items cohered sufficiently for total-score interpretation. Exploratory factor analysis/principal-component extraction supported a dominant single-factor solution, with KMO = 0.925, Bartlett's test of sphericity χ^2^(36) = 1789.589, *P* < 0.001, first-factor variance explained = 79.5%, and item loading range = 0.843–0.943. Because the scale was adapted for the present context, these results and the reliability estimate are reported as preliminary psychometric evidence rather than as definitive validation of a standalone instrument. The scale demonstrated excellent internal consistency in the present study, with a Cronbach's alpha coefficient of 0.967, where a higher total score serves as a global indicator of the extent to which students have internalized the “soft skills” training as a valuable professional resource.

Perceived Barriers Scale (PB). Perceived barriers are conceptualized as situational or psychological obstacles that hinder student engagement and effective learning. In the specific context of medical education, the conflict between “hard sciences” and “soft skills” often leads to the marginalization of humanities curricula, creating unique structural impediments (Hafferty, 1998; Moniz et al., 2021). To measure these challenges, a Learning Barriers Scale was developed based on a review of relevant literature and specific issues within Chinese medical education (Hong et al., 2025; Qian et al., 2018; Zhai et al., 2025; Zhang et al., 2023). Following expert validation and pilot testing, the instrument consists of 6 items structured to assess obstacles across three key domains: Curriculum Conflicts (e.g., time competition with core “hard sciences”), Instructional Quality (e.g., abstract content disconnected from clinical practice), and Evaluation System Flaws (e.g., lack of utility for licensing exams). Responses were recorded on a 5-point Likert scale (1 = strongly disagree, 5 = strongly agree). Because the present study used the perceived barriers items as a global structural-barrier score, we evaluated their coherence for total-score interpretation. Exploratory factor analysis/principal-component extraction supported a dominant single-factor solution, with KMO = 0.909, Bartlett's test of sphericity χ^2^(15) = 1221.217, *P* < 0.001, first-factor variance explained = 85.4%, and item loading range = 0.901–0.942. As with the perceived value measure, this evidence should be interpreted as preliminary support for use in the present exploratory study, and further confirmatory validation in larger multicenter samples is warranted. The reliability of this scale was high, yielding a Cronbach's alpha of 0.966. A higher aggregate score on this scale indicates a stronger resistance to the curriculum, driven by perceived systemic and utilitarian impediments. In this study, structural barriers therefore refer to institutionally embedded constraints on engagement with medical humanities, rather than to a simple lack of student interest. Item refinement emphasized conceptual relevance to structural barriers, clarity of wording, and coverage of both institutional and utilitarian obstacles to engagement.

Academic Burnout Scale (AB). Academic burnout is characterized as a negative psychological syndrome involving emotional exhaustion, cynicism (depersonalization), and a sense of reduced personal accomplishment resulting from chronic academic pressure. In this study, burnout levels were evaluated using a modified short version of the Learning Burnout Scale for Undergraduates originally developed by Lian et al. (2005). This scale contains 6 items stratified into three distinct dimensions: Exhaustion (items 1–2), Improper Behavior/Cynicism (items 3–4), and Reduced Personal Accomplishment (items 5–6). Each item is rated on a 5-point Likert scale. Notably, to ensure measurement validity, the two items assessing reduced personal accomplishment (e.g., “I can effectively solve problems in my studies”) were reverse-scored. Consequently, a higher total score signifies a greater degree of academic burnout. The scale showed acceptable reliability in this study, with a Cronbach's alpha coefficient of 0.734 (Taber, 2018). The lower reliability relative to the two appraisal scales was considered when interpreting associations involving burnout dimensions.

### 2.3 Data processing

We first employed data screening procedures to ensure the validity and reliability of the dataset. Specifically, rigorous exclusion criteria were applied to remove participants from non-clinical majors to ensure sample homogeneity, and to eliminate responses characterized by “straight-lining” patterns (zero variance) to control for low-quality engagement. Subsequently, data management and statistical analysis were performed using SPSS Statistics version 26.0 (IBM Corp., Armonk, NY, USA).

Descriptive statistics, including frequencies, means, and standard deviations, were calculated to summarize demographic characteristics and the distribution of study variables. Preliminary independent *t*-tests were conducted to assess whether key psychological constructs differed by demographic factors (e.g., grade level). Pearson's correlation coefficients were computed to examine bivariate associations between medical humanities cognition and academic burnout variables. Finally, to provide a robust understanding of the multivariate relationship between the “cognition” variable set (Perceived Value, Perceived Barriers) and the “burnout” variable set (Exhaustion, Cynicism, Academic Efficacy), a Canonical Correlation Analysis (CCA) was employed. This technique was specifically chosen for its ability to analyze the complex structure between two sets of variables simultaneously, rather than examining them in isolation. Statistical significance was defined as a two-tailed *P*-value < 0.05. For CCA, we reported model fit, standardized canonical weights, structure coefficients, cross-loadings, and redundancy indices; interpretation prioritized structure coefficients because they are less unstable than weights when variables within a set are correlated.

Before interpreting the CCA, the correlation matrix was inspected to ensure that the variables were theoretically related but not redundant, and canonical functions were retained only when supported by Wilks' lambda and statistical significance. Because all core variables were collected through self-report questionnaires, the possibility of common method variance and social desirability bias was considered when interpreting the results. The analysis was also interpreted in light of the cross-sectional design, the modest single-institution sample, and the absence of direct adjustment for workload, examination pressure, clinical exposure, socioeconomic background, personality, or baseline mental health.

## 3 Results

### 3.1 Descriptive statistics, demographic influences, and bivariate correlations

Table 1 presents the descriptive statistics for the study variables (*N* = 165). Preliminary independent *t*-tests revealed no significant differences in perceived value, perceived barriers, or academic burnout scores between freshmen and sophomores (*P* > 0.05). Consequently, demographic variables were excluded from subsequent multivariate models to preserve statistical power and focus on the primary psychological constructs. This decision should be understood as an exploratory modeling choice rather than as evidence that demographic or educational confounding was absent.

**Table 1:** Demographic characteristics and descriptive statistics of medical humanities cognition and academic burnout of clinical medical students (*N* = 165).

Variables	Category/range	*N* (%) or *M ±SD*
Demographic characteristics		
Grade	Freshmen	39 (23.6%)
	Sophomores	126 (76.4%)
Medical humanities cognition		
Perceived value	1–5	4.25 ± 0.69
Perceived barriers	1–5	3.06 ± 1.22
Academic burnout		
Emotional exhaustion	1–5	2.70 ± 1.04
Cynicism	1–5	2.15 ± 0.85
Academic efficacy	1–5	3.21 ± 0.87

*M*, mean; *SD*, standard deviation.

**Figure 1:**
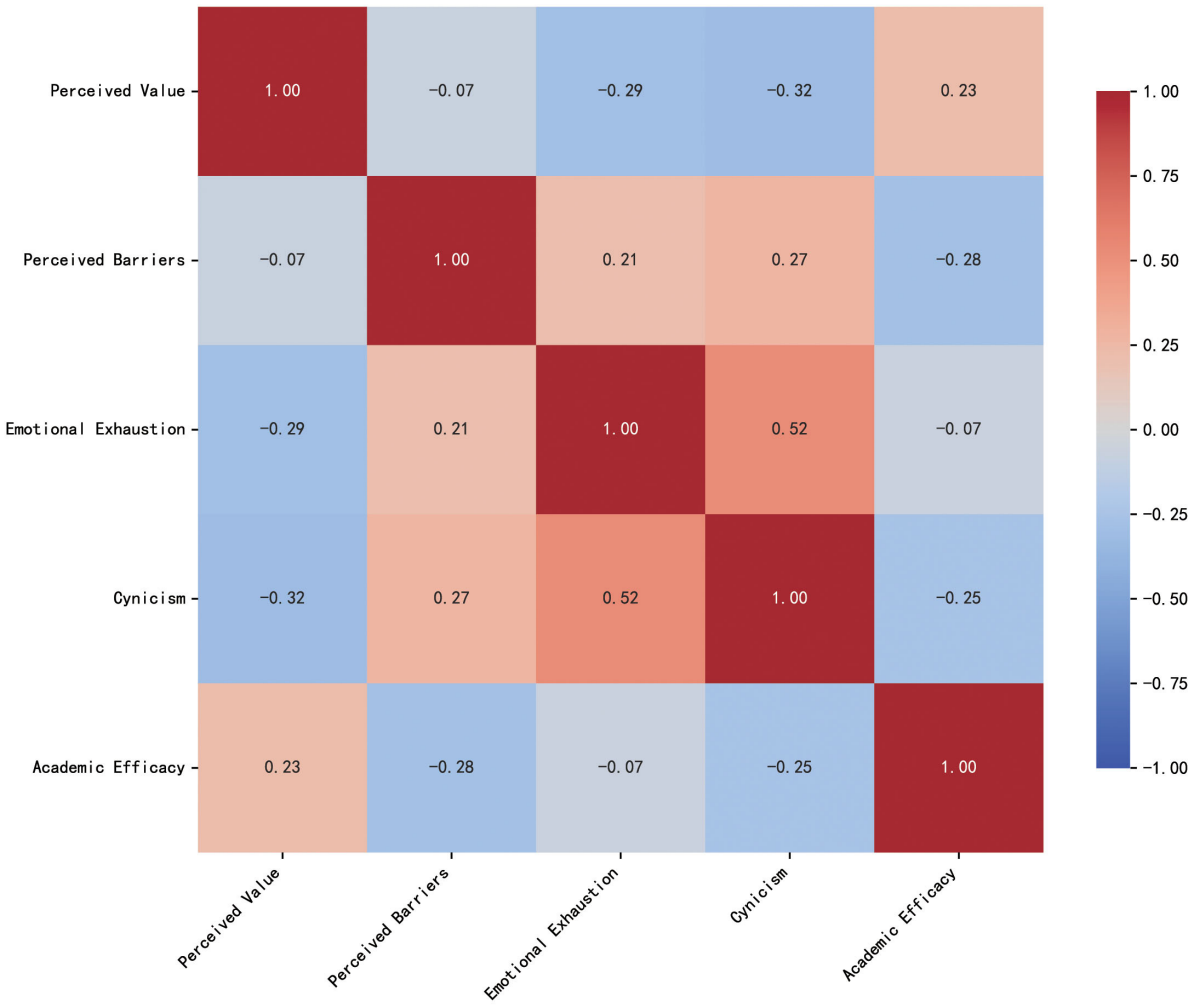
Heatmap visualizing the inter-correlations between medical humanities cognitive appraisals and academic burnout dimensions. Color intensity represents the magnitude of the Pearson correlation coefficient (*r*), ranging from −1 (blue) to +1 (red).

On average, medical students demonstrated a relatively high recognition of the value of medical humanities (*M* = 4.25, *SD* = 0.69), yet they also perceived a moderate level of barriers (*M* = 3.06, *SD* = 1.22). Regarding academic burnout, the mean scores for emotional exhaustion and cynicism were 2.70 and 2.15, respectively. Results from the Pearson correlation analysis, as summarized in Table 2 and visualized in Figure 1, reveal distinct patterns of association among the variables. Perceived Value showed significant negative correlations with both Cynicism (*r* = −0.319, *P* < 0.001) and Emotional Exhaustion (*r* = −0.290, *P* < 0.001), and a positive correlation with Academic Efficacy (*r* = 0.225, *P* < 0.01). Conversely, Perceived Barriers were positively associated with Cynicism (*r* = 0.266, *P* < 0.001) and Exhaustion (*r* = 0.206, *P* < 0.01). As illustrated in Figure 1, Perceived Value and Perceived Barriers showed no significant correlation (*r* = −0.075, *p* > 0.05). This empirical independence indicates that these two cognitive dimensions operate as distinct constructs, where a strong appreciation for humanistic values does not inherently offset the perception of structural obstacles.

**Table 2:** Pearson correlation matrix of perceived value, perceived barriers, and academic burnout variables.

Variable	Perceived value	Perceived barriers	Emotional exhaustion	Cynicism	Academic efficacy
Perceived value	1				
Perceived barriers	−0.075	1			
Emotional exhaustion	−0.290^***^	0.206^**^	1		
Cynicism	−0.319^***^	0.266^***^	0.525^***^	1	
Academic efficacy	0.225^**^	−0.280^***^	−0.069	−0.254^***^	1

^*^P < 0.05. ^**^P < 0.01. ^***^P < 0.001. ^*^ denotes statistical significance; more asterisks mean stronger evidence against the null hypothesis.

### 3.2 Canonical correlation analysis (CCA)

To explore the multivariate relationship between students' cognitive appraisals and academic burnout, we conducted a canonical correlation analysis. The two dimensions of medical humanities cognition (Perceived Value, Perceived Barriers) were designated as the cognition variable set, while the three dimensions of burnout (Emotional Exhaustion, Cynicism, Academic Efficacy) served as the burnout variable set.

The analysis identified two pairs of canonical variates. The first **canonical variate pair** was statistically significant (*r*_*c*_ = 0.502, *r*_*c*_^2^ = 0.252, Wilks' λ = 0.743, χ^2^ = 47.765, *df* = 6, *P* < 0.001), indicating a moderate multivariate association between the cognition set and the burnout set. The second variate pair was not significant (*r*_*c*_ = 0.076, *r*_*c*_^2^ = 0.006, Wilks' λ = 0.994, χ^2^ = 0.942, *df* = 2, *P* = 0.624); thus, the interpretation focused solely on the first pair.

To avoid conflating canonical weights and structure coefficients, Table 3 now reports both statistics for the first canonical function. The first function explained 25.2% of the shared variance (*r*_*c*_^2^ = 0.252). In the cognition set, standardized canonical weights were 0.711 for Perceived Value and −0.652 for Perceived Barriers; the corresponding structure coefficients were 0.760 and −0.705, and the cross-loadings were 0.382 and −0.354. In the burnout set, standardized canonical weights were −0.408 for Emotional Exhaustion, −0.445 for Cynicism, and 0.541 for Academic Efficacy; the corresponding structure coefficients were −0.678,−0.796, and 0.682, with cross-loadings of −0.341, −0.400, and 0.343. Redundancy indices indicated that the first burnout variate explained 13.6% of variance in the cognition set, whereas the first cognition variate explained 13.1% of variance in the burnout set.

Table 3Canonical correlation analysis results between medical humanities cognition and academic burnout.

**Table:** 

Panel A. Model summary								
Function	*r_*c*_*	*r_*c*_^2^*	*Wilks' λ*	*χ^2^*	*df*	*P*	Cognition-setredundancy	Burnout-set redundancy
1	0.502	0.252	0.743	47.765	6	< 0.001	0.136	0.131
2	0.076	0.006	0.994	0.942	2	0.624	0.003	0.002

**Table:** 

Panel B. Coefficients for the first canonical function				
Variable set	Variable	Standardized canonical weight	Structure coefficient/ loading	Cross-loading
Cognition	Perceived value	0.711	0.760	0.382
Cognition	Perceived barriers	−0.652	−0.705	−0.354
Burnout	Emotional exhaustion	−0.408	−0.678	−0.341
Burnout	Cynicism	−0.445	−0.796	−0.400
Burnout	Academic efficacy	0.541	0.682	0.343

*r*_*c*_, canonical correlation; *r*_*c*_^2^, squared canonical correlation. Structure coefficients are canonical loadings. Cross-loadings are correlations between each observed variable and the opposite canonical variate. Redundancy indices indicate the proportion of variance in one variable set explained by the opposite canonical variate. Only the first canonical function was statistically significant and was therefore retained for interpretation.

**Figure 2:**
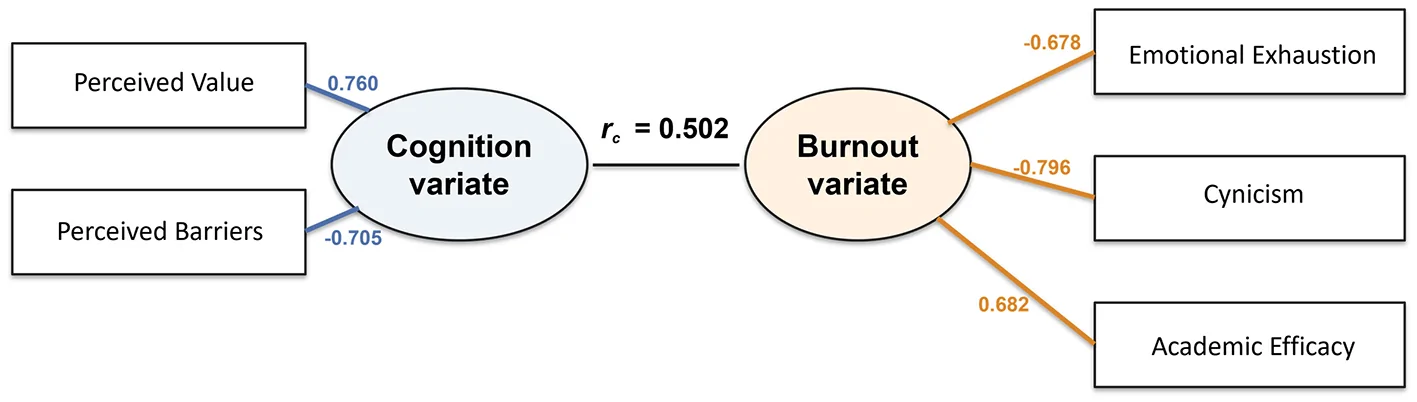
Path diagram of the first canonical correlation function between medical humanities cognition and academic burnout. Values adjacent to observed variables represent structure coefficients/canonical loadings rather than standardized canonical weights. The canonical correlation was *r*_*c*_ = 0.502. Full canonical weights, cross-loadings, significance tests, and redundancy indices are reported in Table 3.

As visualized in Figure 2, the significant first function (*r*_*c*_ = 0.502) suggests an associative pattern in which students with higher perceived value and lower perceived barriers tended to report lower emotional exhaustion and cynicism and higher academic efficacy. Because Cynicism had the largest absolute structure coefficient within the burnout set, the value-barrier profile appeared most strongly related to psychological detachment; however, this interpretation remains correlational and should not be read as evidence that structural barriers cause cynicism.

## 4 Discussion and conclusion

The present study reveals a distinctive “cognitive paradox” among Chinese clinical medical students under the pervasive “hard science vs. soft skills” tension: they maintain high Perceived Value for medical humanities (*M* = 4.25) while simultaneously reporting moderate Perceived Barriers (*M* = 3.06). This finding explicitly addresses the cognitive tension between the rigorous demands of biomedical training and the internal drive for humanistic education. This suggests that students have internalized the necessity of the biopsychosocial model, contrasting with earlier concerns that “soft skills” might be dismissed as secondary in technically driven environments. However, the persistence of structural barriers—specifically time conflicts and formalistic assessments—indicates that the pedagogical challenge has shifted from “ideological acceptance” to “structural feasibility.” This aligns with Hidden Curriculum Theory (Hafferty, 1998), which posits that the implicit values conveyed by an intense biomedical workload and rigid institutional structures may undermine the explicit humanistic goals of the formal curriculum (Iorio et al., 2022).

### 4.1 The “hard science vs. soft skills” paradox: cognitive decoupling

A pivotal and novel finding of this study is the non-significant correlation between Perceived Value and Perceived Barriers (*r* = −0.075, *p* > 0.05). This empirical evidence challenges the traditional pedagogical assumption that high perceived importance naturally motivates students to overcome obstacles. From the perspective of the Job Demands-Resources (JD-R) model (Bakker and Demerouti, 2017), Perceived Value functions as a motivational “resource”, while Perceived Barriers act as a “demand”. Our results indicate a state of “cognitive decoupling” between these two constructs. This suggests that students process the “moral worth” of humanities and the “structural cost” of learning them in separate cognitive tracks. Consequently, educational interventions focusing solely on “value persuasion” (increasing resources) may not automatically reduce the structural friction (reducing demands). This insight aligns with Expectancy-Value Theory (EVT), which emphasizes that task value and cost are distinct components of the motivational calculus; high value does not nullify high cost (Eccles and Wigfield, 2020). This interpretation is also consistent with recent work on motivation, engagement, and burnout, which emphasizes that motivational resources and burnout-related outcomes are connected through complex psychological pathways rather than through a single linear mechanism (Zeng et al., 2025).

### 4.2 The mechanism of cynicism: structural barriers and psychological detachment

The Canonical Correlation Analysis (CCA) provides a nuanced understanding of how these cognitive appraisals relate to multidimensional burnout. Specifically, our analysis helps interpret why cynicism was the most prominent burnout dimension in the multivariate association pattern. The structure coefficients revealed that the “High Value—Low Barriers” profile showed its strongest relation to reduced Cynicism (loading = −0.796), surpassing its association with Emotional Exhaustion (−0.678).

By inverse implication, this mathematical asymmetry suggests that high structural friction may be particularly relevant to cynical attitudes. This finding suggests that structural barriers were more closely associated with defensive psychological detachment than with physical fatigue alone, consistent with the conceptual core of cynicism (Maslach and Leiter, 2021). This structural weight of cynicism aligns with Psychological Contract Theory. When medical training fails to reconcile students' humanistic ideals with the rigid efficiency demands of the biomedical curriculum, a “breach” may occur. Students who experience this misalignment may be more likely to report depersonalized or cynical attitudes, although longitudinal data are needed to test this proposed pathway (Liu et al., 2023).

### 4.3 Perceived value as a cognitive resource associated with burnout

Beyond the detrimental associations of structural barriers, our findings clarify the role of “Perceived Value” as a cognitive appraisal associated with student burnout. Within the multivariate framework, perceived value was associated with a more favorable burnout profile and may represent a cognitive resource. When students strongly internalize the ethical and practical significance of medical humanities, this cognitive appraisal may be linked to lower emotional exhaustion and sustained academic efficacy. However, this association appears conditional on the institutional context in which humanities education is delivered. High perceived value alone may not be sufficient when students simultaneously experience overwhelming structural constraints. These findings suggest that institutional reforms should address these barriers when attempting to strengthen the potential educational benefits of humanities training.

### 4.4 Implications for medical education reform

Since cynicism is associated with the erosion of empathy (Chunming et al., 2017; Wang et al., 2023), these structural impediments ironically may contribute to the very “loss of empathy” that humanities education is intended to prevent. Based on our findings, we propose a paradigm shift from an “additive” to an “integrative” curriculum reform. To address barriers associated with cynicism in the present analysis, institutions should move beyond the “siloed” teaching model. Drawing on Situated Learning Theory, humanistic training should be infused into existing clinical clerkships through methods like bedside ethics rounds (Iorio et al., 2022). This integration may minimize time competition with biomedical studies while bridging the gap between humanistic theory and clinical practice (Wald, 2020). Furthermore, shifting from formalistic tests to clinically integrated evaluations could lower the perceived “burden” of the curriculum, fostering a learning environment that supports both academic efficacy and professional resilience.

For example, humanities learning can be embedded into problem-based learning cases, standardized-patient communication training, and objective structured clinical examination stations, where ethical reasoning, empathy, and communication are assessed alongside biomedical reasoning. Digital support services may also help institutions deliver flexible learning resources and feedback without simply adding more classroom hours (Subbarao et al., 2025). These examples suggest that the integrative model can be adapted to different institutional contexts according to curriculum capacity and assessment structures. This implementation logic is also consistent with recent curricular-model literature emphasizing longitudinal integration, structured reflection, and explicit evaluation rather than isolated humanities activities (Coronado-Vazquez et al., 2025).

### 4.5 Limitations and strengths

Several limitations should be noted. First, the cross-sectional design precludes causal inferences. Second, all data were collected via self-report questionnaires, which introduces potential common method variance and social desirability bias—particularly given the socially expected nature of expressing positive attitudes toward medical humanities. Third, the sample size is relatively small (*N* = 165) and restricted to a single Chinese medical institution, which may limit broader generalizability. However, the selected institution provides a highly relevant context for this exploratory study. As an “applied” medical university dedicated to training practitioners for grassroots healthcare, its curriculum is heavily oriented toward practical clinical skills. In such a pragmatism-driven educational environment, the tension between rigorous biomedical demands and humanistic ideals—the “hard science vs. soft skills” paradox—is particularly pronounced. Therefore, this specific setting offers a focused lens to examine the structural friction and cognitive decoupling among a critical segment of the medical student population. Future multicenter studies across diverse academic tiers and cultural contexts are required to validate these findings. Additionally, longitudinal research is needed to track how the “Value-Barrier” dynamic evolves as students transition from preclinical years to high-pressure clinical practice. Despite these limitations, the study's strengths include the utilization of Canonical Correlation Analysis (CCA), which provides a holistic understanding of the multivariate relationship between cognition and burnout, surpassing the limitations of isolated univariate analysis. By deconstructing the “cognitive paradox” between value and barriers, this study offers fresh empirical evidence for the “hard science vs. soft skills” dilemma, providing a data-driven foundation for structural integration in medical education.

Furthermore, this study did not directly account for several potential confounding variables, including academic workload, examination pressure, clinical exposure, socioeconomic background, personality traits, and baseline mental health. Moreover, because the sample consisted predominantly of first- and second-year students, the findings may not fully reflect how perceptions of medical humanities and burnout evolve during clinical rotations—a period when patient contact and professional identity formation become more salient. Future research should therefore employ larger multicenter cohorts, longitudinal designs, and multi-method data collection (e.g., qualitative interviews, behavioral metrics, and clinical evaluations) to test whether these associations remain stable across different training stages and institutional contexts.

## 5 Conclusion

In conclusion, this study reveals a critical “cognitive decoupling” within medical training: students' high valuation of humanities is psychologically distinct from the structural burdens they experience. Our findings highlight that structural friction is closely linked to cynical detachment among medical students. Therefore, future reforms should shift from mere ideological promotion to structural embedding, where humanities are integrated into clinical practice not only for pedagogical coherence but also as a potential way to reduce perceived cognitive costs. Within the limits of a single-center cross-sectional study, these findings suggest that addressing structural conflict may help medical education better support the empathetic resilience of the future medical workforce.

## Data Availability

The original contributions presented in the study are included in the article/supplementary material, further inquiries can be directed to the corresponding author.
